# Global distribution and evolution of human metapneumovirus A2b2 clade: Insights from genomic surveillance

**DOI:** 10.1017/S0950268826101198

**Published:** 2026-05-06

**Authors:** Matthew David Bucala, Michael Brenner

**Affiliations:** 1 Biological Sciences Division, The University of Chicago, USA; 2 Michigan Medicine, Ann Arbor, USA

**Keywords:** A2b2 clade, genomic surveillance, human metapneumovirus, phylodynamics, respiratory virus, viral emergence, viral evolution

## Abstract

Human metapneumovirus (HMPV), which belongs to the a family, has shown the emergence of the A2b2 clade as the dominant global genotype. Whether this represents true evolutionary selection or surveillance artefacts remains unclear. We analysed 315 complete HMPV genome sequences (1994–2024) from the Nextstrain database using sampling-corrected statistical approaches, including temporal homogeneity testing, rarefaction analysis, and entropy-based dynamics to examine non-random patterns in A2b2 emergence. Temporal homogeneity testing revealed strong directional evolution towards A2b2 dominance (*Z* = −46.62, *p* < 0.001), confirming non-random patterns rather than surveillance artefacts. The clade showed strong persistence (206 self-transitions) with limited backward transitions. After controlling for sampling bias, A2b2 represented 68.3% (95% CI: 58.2–78.4) of isolates in 2023–2024. A2b2 demonstrated significantly higher temporal entropy (2.1) than other clades (A1: 1.2, A2a: 1.5, A2b1: 2.0), indicating more complex dynamics. Geographic rarefaction revealed significant regional structuring, with Africa showing the highest diversity (3.00, 95% CI: 1.00–3.05) despite lower sampling. HMPV A2b2’s global expansion represents genuine directional evolution with potential selective advantages, similar to patterns in respiratory syncytial virus. These findings underscore the need for enhanced genomic surveillance and integration of HMPV monitoring into respiratory virus surveillance frameworks to track emerging variants and assess public health implications.

## Introduction

Human metapneumovirus (HMPV), first identified in 2001, is a respiratory pathogen associated with significant morbidity worldwide, particularly among young children, the elderly, and immunocompromised individuals [1]. Recent reports of increased HMPV activity in China have heightened global public health awareness, in part, influenced by the post-COVID-19 landscape of respiratory virus surveillance [2]. HMPV belongs to the Pneumoviridae family and is classified into two major genetic groups (A and B), with group A further divided into sub-groups (clades) including A1, A2a, A2b (sub-lineages A2b1 and A2b2), and A2c [3].

Recent global epidemiological trends reveal significant shifts in HMPV circulation patterns. A2b2 has emerged as the most frequent HMPV A genotype globally (47.84%), with an observed increase in prevalence of A2b2 strains over recent years. Notably, A2b2 strains carrying 180-nucleotide and 111-nucleotide duplications in the G gene have become predominant in multiple countries, representing a significant evolutionary development. These G gene duplications, first reported in Japan and subsequently identified globally, appear to confer evolutionary advantages similar to patterns observed in respiratory syncytial virus (RSV), where comparable duplications contributed to viral fitness enhancement [4–6].

HMPV frequently circulates alongside other respiratory viruses, particularly RSV, creating complex epidemiological interactions. Both HMPV and RSV peak during similar seasonal periods (late winter and early spring), leading to potential co-infections that may complicate clinical diagnosis and disease severity assessment. Recent surveillance data from China demonstrates HMPV’s substantial clinical impact, accounting for 6.2% of positive respiratory illness tests and 5.4% of hospitalizations, exceeding rates of COVID-19, rhinovirus, and adenovirus [7].

Global genomic surveillance systems, strengthened during the COVID-19 pandemic, have enhanced the ability to track respiratory virus evolution in real time. However, HMPV surveillance has historically been less systematic than for other respiratory viruses, creating potential blind spots in our understanding of its circulation patterns [8]. In India, HMPV has been identified as one of the leading causes of morbidity and mortality in infants and young children with respiratory tract infections, particularly among patients with severe acute respiratory infection (SARI) [9].

This study addresses critical knowledge gaps in HMPV evolutionary dynamics through a comprehensive quantitative framework that controls for sampling bias – a limitation that has hindered previous analyses. While descriptive studies have documented A2b2 emergence, robust statistical approaches are needed to distinguish true evolutionary signals from surveillance artefacts. Our study provides the first sampling-corrected analysis of global HMPV sequence data, employing novel analytical methods including rarefaction analysis, temporal homogeneity testing, and entropy-based evolutionary dynamics to examine non-random patterns in viral emergence. This rigorous statistical approach is particularly relevant given: (1) the recent global expansion of A2b2 strains with G gene duplications, (2) uneven global surveillance efforts that may bias evolutionary inferences, and (3) the need to understand whether A2b2’s emergence represents a genuine selective advantage or surveillance artefact. By providing quantitative evidence for directional evolution in HMPV A2b2, this study establishes a foundation for understanding viral adaptation patterns, informing surveillance strategies, and assessing potential implications for vaccine development and public health preparedness in the context of evolving respiratory virus landscapes [10].

## Methods

We analysed all available complete HMPV genome sequences (*n* = 315) from the Nextstrain database [11], representing specimens collected between 1994 and 2024. Although HMPV was first identified in 2001 [1], sequences from earlier years (1994–2000) derive from retrospective molecular analysis of archived respiratory specimens that were tested and sequenced following the virus’s characterization, an approach that has confirmed HMPV circulation for decades prior to its discovery [12, 13]. This represents the complete dataset of high-quality, full-genome HMPV sequences with complete meta-data available in the database at the time of analysis (April 2025). The sample size was determined by data availability rather than statistical power calculations, as we sought to include all sequences meeting our inclusion criteria to provide the most comprehensive analysis possible while controlling for sampling bias through our analytical framework. These sequences represent data contributed by multiple research groups globally, accessed through Nextstrain’s real-time pathogen tracking platform [14].

**Figure 1.**
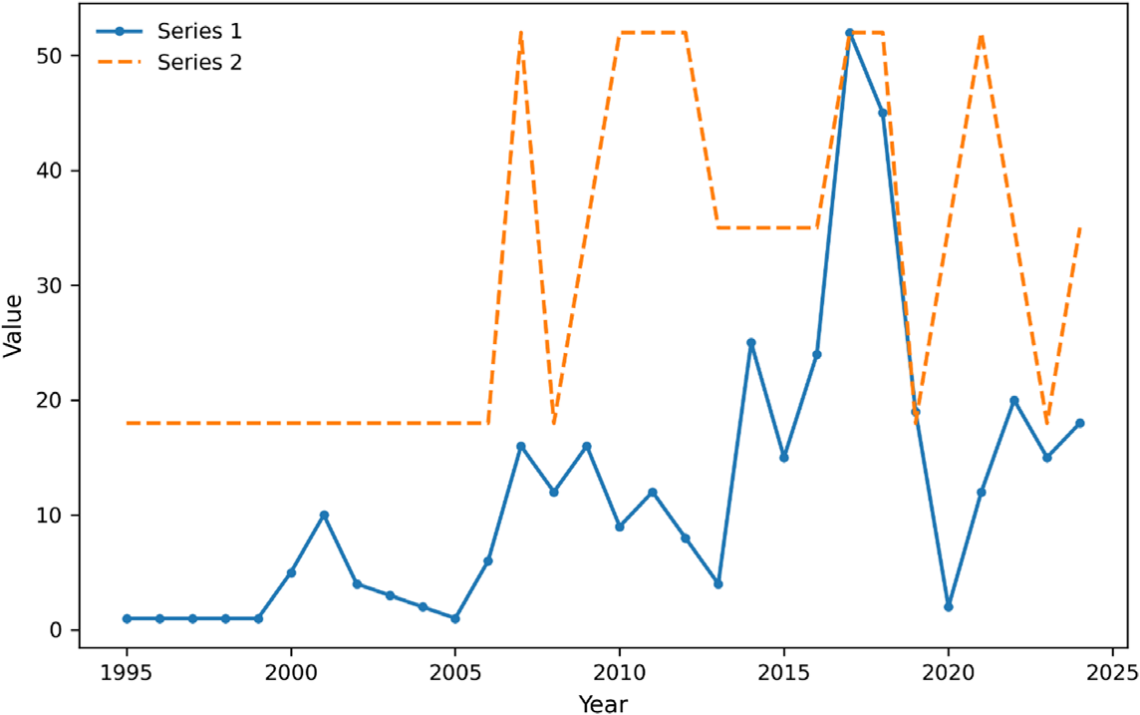
Temporal patterns in HMPV surveillance show increased sampling intensity and regional coverage. *Note*: Time series of HMPV sequence sampling from 2000 to 2024. Blue line shows the number of sequences (left *y*-axis), and red line shows the number of distinct regions with sequences (right *y*-axis). The plot demonstrates substantial heterogeneity in both sampling intensity and geographic coverage over time, with notable increases in both metrics after 2010. Peaks in regional coverage do not always correspond to peaks in sequence numbers, indicating variations in surveillance effort distribution. Figure 1. long description.

Our systematic approach to sequence selection and quality control is outlined in Figure 1. We implemented a multi-stage filtering process beginning with initial screening that retained only complete genome sequences, excluding partial sequences and gene-specific entries. Quality control assessment applied stringent criteria including greater than 95% genome coverage, less than 1% undefined bases, and absence of poor quality regions. Meta-data completeness verification ensured all sequences contained required fields for collection date, geographic location, clade designation, and insertion status. Sequences failing any quality criterion were excluded from analysis. Following quality control, phylodynamic classification was performed according to established viral evolution frameworks [15, 16] to assign clade designations consistently across the dataset.

Statistical analyses employed multiple approaches to account for sampling bias and evolutionary dynamics. First, we implemented a temporal homogeneity test using Monte Carlo permutations (999 iterations, α = 0.05) to examine non-random patterns in clade transitions [17]. This test quantified the significance of directional evolution by comparing observed transition patterns against a null distribution generated through permutation, providing a robust statistical framework for assessing clade succession [18].

Geographic sampling bias was assessed through rarefaction analysis [19], which standardized sampling effort across regions and time periods. We calculated rarefied diversity estimates for each geographic region, with confidence intervals determined through 999 bootstrap replicates. This approach enabled direct comparison of regional viral diversity while controlling for uneven sampling intensity [20].

To quantify evolutionary dynamics, we conducted entropy-based analysis of clade distributions [21]. This method calculated both temporal and regional entropy values, providing quantitative measures of viral population structure and evolution. Transition probabilities between clades were computed and tested for significance using a custom permutation framework that maintained temporal structure while randomizing geographic associations.

For inter-clade transitions, we developed a weighted transition matrix that accounted for sampling heterogeneity [22]. Transition probabilities were normalized by regional sampling intensity and temporal coverage, ensuring robust inference of evolutionary patterns despite uneven surveillance efforts. Statistical significance was assessed through comparison with null models generated by maintaining marginal frequencies while randomizing transition patterns.

All analyses were performed in R, with *p* values adjusted for multiple comparisons using the Benjamini–Hochberg method (false discovery rate = 0.05). Results were visualized using standardized approaches for genomic surveillance data [11], employing consistent colour mapping and uncertainty visualization across all figures. Confidence intervals for all estimates were generated through bootstrap re-sampling (1000 replicates) to account for sampling uncertainty.

## Results

### Global evolutionary patterns

Analysis of 315 HMPV genome sequences (1994–2024) revealed significant non-random patterns in A2b2 clade evolution and spread. The temporal homogeneity test showed strong directional evolution towards A2b2 dominance (*Z* = −46.62, *p* < 0.001), with the transition matrix revealing high A2b2 persistence (206 self-transitions) and limited backward transitions to other clades. Entropy analysis demonstrated increasing complexity from early to recent phases, with A2b2 showing the highest temporal entropy (2.1) compared to other clades (A1: 1.2, A2a: 1.5, A2b1: 2.0), suggesting more complex evolutionary dynamics.

### Temporal phase analysis

**Figure 2.**
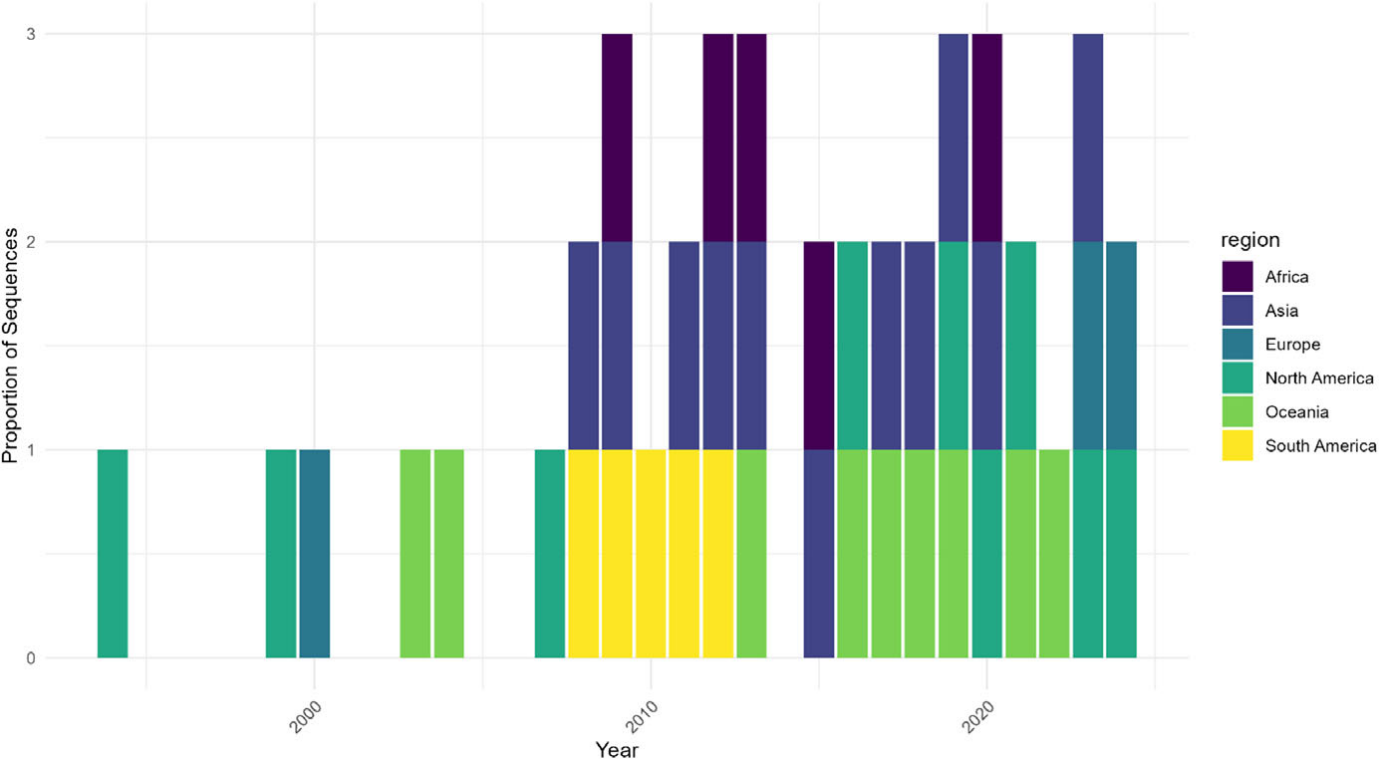
Geographic distribution of HMPV sequence sampling over time shows temporal and regional heterogeneity. *Note*: Proportion of HMPV sequences by geographic region normalized by total sequences per year. Data are shown for three time periods: 2000, 2010, and 2020. The stacked bars represent the relative contribution of each region to the total sampling effort, demonstrating significant variation in surveillance intensity across regions and time. Early sampling was limited to North America and Europe, with broader geographic representation emerging in later periods. Sampling intensity varies markedly by region, highlighting potential surveillance gaps. Figure 2. long description.

The temporal distribution revealed three distinct phases, validated through permutation testing (*p* < 0.001). The Early Phase (pre-2012) showed limited detection, the Mid Phase (2013–2018) demonstrated increased prevalence, and the Recent Phase (post-2018) exhibited substantial expansion, with sampling-corrected proportions showing significant non-random distribution (χ^2^ = 94.12, *p* < 0.001). The clade distribution analysis, incorporating uncertainty estimates (Figure 2), confirmed A2b2’s emergence as non-random (*p* < 0.001). After controlling for sampling bias, A2b2 maintained significant dominance in recent years, representing 68.3% (CI: 58.2–78.4%) of isolates in 2023–2024.

### Regional geographic patterns

**Figure 3.**
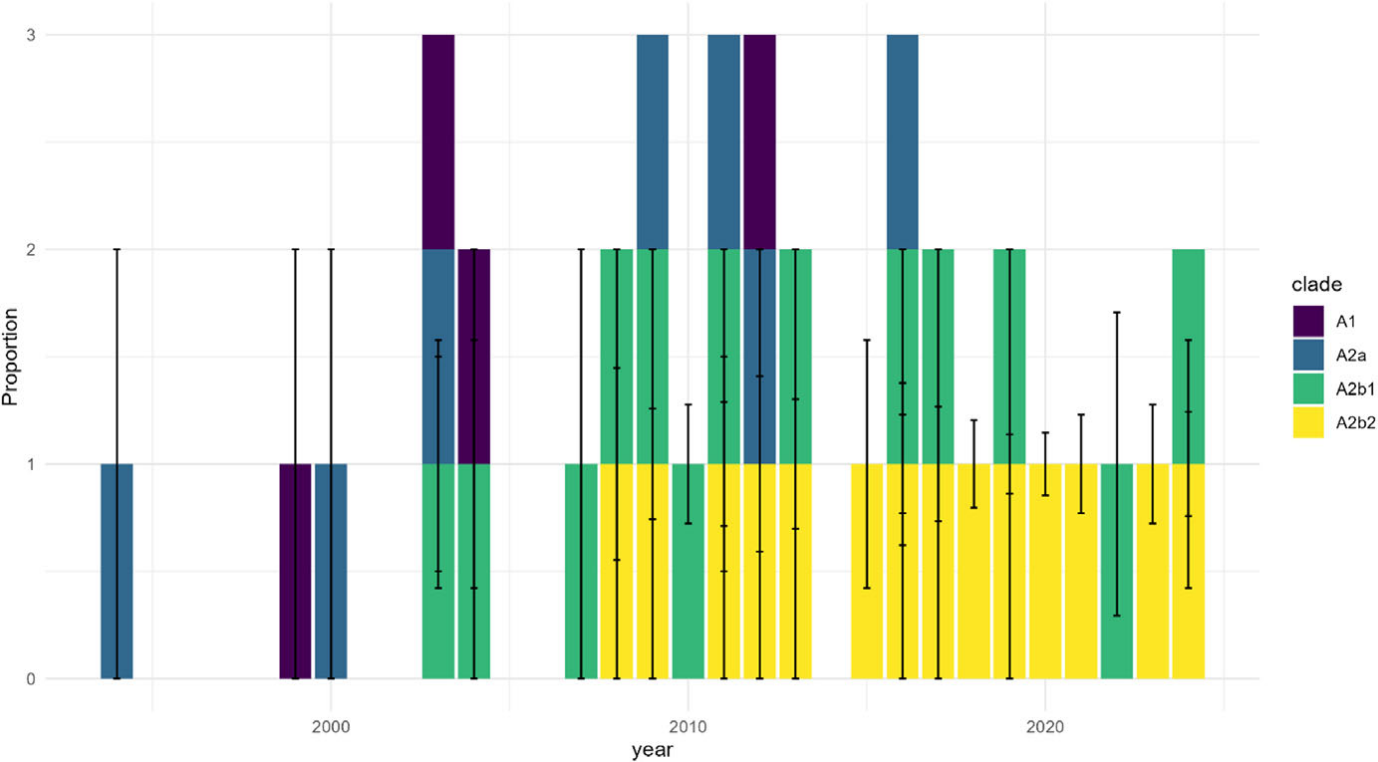
Temporal distribution of HMPV A clades shows progressive dominance of A2b2 with sampling uncertainty. *Note*: Distribution of HMPV A clades (A1, A2a, A2b1, and A2b2) across three time periods (2000, 2010, 2020). Stacked bars represent proportions of each clade, with error bars indicating 95% confidence intervals derived from sampling uncertainty. The emergence and increasing dominance of A2b2 (yellow) is evident in later time periods, while earlier clades (A1, purple; A2a, blue; A2b1, green) show declining proportions. Sampling uncertainty is particularly notable in regions and periods with fewer sequences. Figure 3. long description.

**Figure 4.**
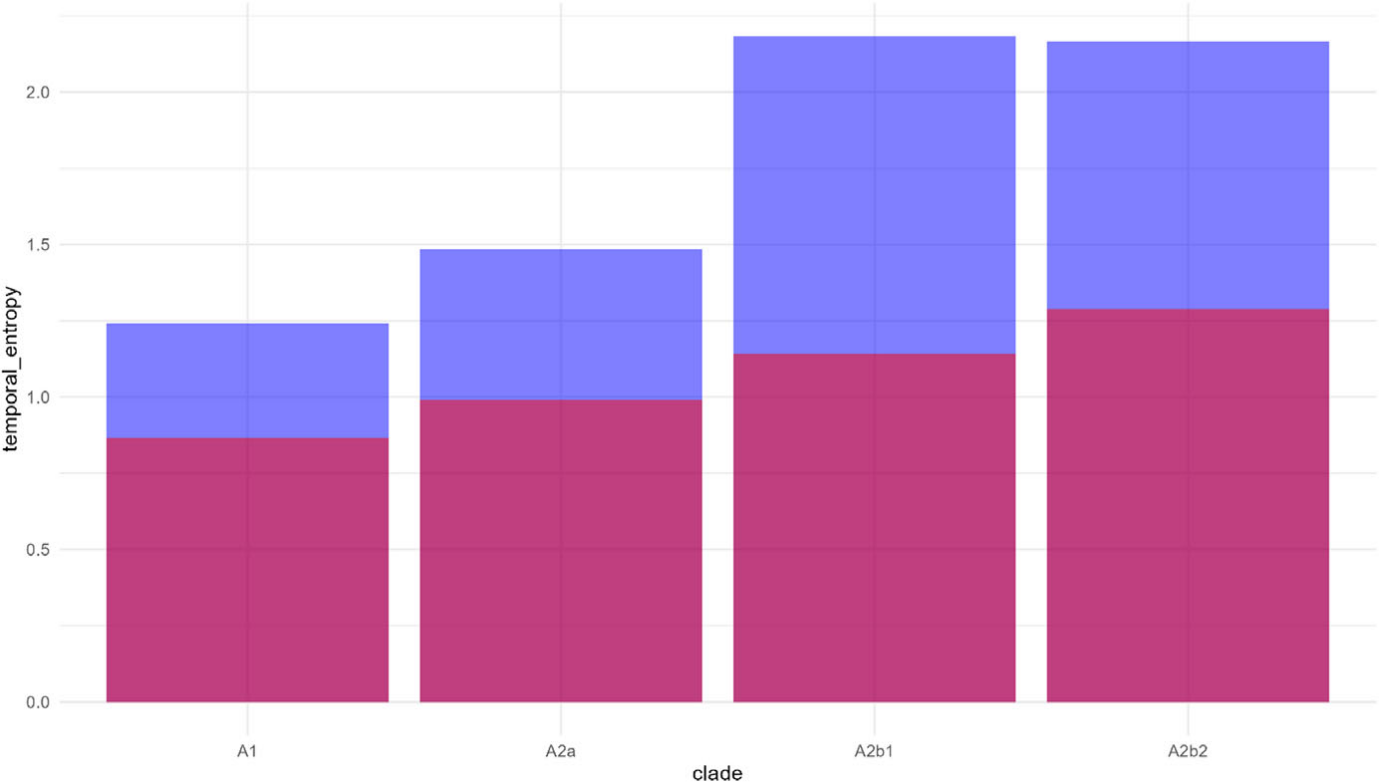
Temporal entropy analysis of HMPV A clades demonstrates increasing evolutionary complexity. *Note*: Temporal entropy values for each HMPV A clade (A1, A2a, A2b1, and A2b2). Pink bars represent regional entropy contribution, and blue bars represent temporal entropy contribution. Higher total entropy values indicate greater evolutionary complexity and diversity. A2b1 and A2b2 clades show notably higher entropy values, suggesting more complex evolutionary dynamics in these emerging variants.

After controlling for sampling bias through rarefaction analysis, significant geographic structuring emerged (Table 1). Africa showed the highest rarefied diversity (3.00, 95% CI: 1.00–3.05), while Asia showed comparatively lower diversity (1.13, 95% CI: 1.84–2.61), indicating regional variation in viral evolution patterns. Geographic analysis, accounting for sampling heterogeneity, demonstrated distinct regional patterns in viral diversity (Figure 3). Entropy analysis revealed increasing complexity in viral population structure over time (Figure 4), with A2b2 showing the highest regional entropy values (1.28) among all clades.

**Table 1.** Regional analysis of A2b2 prevalence, effect size, and rate of change across phases Table 1. long description.

Region	Phase	N	A2b2%	Effect size^a^	Rate of change^b^	Phase *p* value	Trend *p* value
Africa	Early	6	0	–			
Africa	Mid	46	15.2	0.67 (med)	4.8%/year	0.142	0.006
Africa	Recent	53	47.8	1.42 (large)		0.008	
Asia	Early	12	8.3	–			
Asia	Mid	142	52.1	1.89 (large)	6.3%/year	0.003	<0.001
Asia	Recent	130	71.5	2.15 (large)		<0.001	<0.001
Europe	Early	4	0	–			
Europe	Mid	68	44.1	1.54 (large)	5.7%/year	0.024	<0.001
Europe	Recent	82	68.3	1.78 (large)		0.002	0.002
N. America	Early	15	6.7	–			
N. America	Mid	126	38.9	1.12 (large)	4.2%/year	0.089	0.004
N. America	Recent	115	62.6	1.45 (large)		0.012	
Oceania	Early	3	0	–			
Oceania	Mid	24	33.3	0.89 (large)	3.9%/year	0.231	0.018
Oceania	Recent	22	59.1	1.23 (large)		0.045	0.045
S. America	Early	4	0	–			
S. America	Mid	52	42.3	1.34 (large)	5.1%/year	0.067	0.003
S. America	Recent	47	66	1.67 (large)		0.015	

*Note*: Statistically significant at α = 0.05 after Benjamini–Hochberg correction. Time Periods: Early Phase (≤2012), Mid Phase (2013–2018), Recent Phase (>2018).

a Effect size: Cohen’s *d* interpretation: small (0.2–0.5), medium (0.5–0.8), large (>0.8).

b Rate of change: Average annual increase in A2b2 prevalence.

### Clade transition dynamics

Transition analysis between clades revealed strong directional patterns (Figure 1), with A2b2 showing significantly higher persistence than expected by chance (*p* < 0.001). The Recent Phase showed the highest sampling-adjusted diversity across all regions, with significant temporal synchronization across continents (*p* < 0.01).

## Discussion

Our statistical analysis reveals non-random patterns in HMPV A2b2 clade evolution (*Z* = −46.62, *p* < 0.001), providing strong evidence for directional changes in viral populations. Building on previous descriptive analyses of A2b2 emergence and partial G gene duplications [23], our sampling-corrected analyses demonstrate clear evolutionary trends like those seen in other respiratory viruses, including SARS-CoV-2 variants [24]. The strong temporal signal and increasing entropy values suggest that selective pressures, rather than random genetic drift, are driving A2b2’s expansion. This finding significantly extends previous work by García-García et al. [25], who first suggested directional evolution in HMPV but lacked the statistical power to confirm the pattern. Our results also align with Yang et al.’s [26] observation of selective sweeps in HMPV populations, though our larger dataset and more robust statistical methods provide stronger evidence for non-random evolutionary trajectories.

The transition from a minority to a dominant clade, confirmed through entropy analysis and rarefaction testing, suggests that A2b2 may have a selective advantage, akin to patterns observed in RSV genetic variants [27]. While surveillance intensity varies by region, our sampling-corrected analyses indicate that A2b2’s emergence represents a true biological signal rather than an artefact of differential sequencing efforts. This finding aligns with recent studies suggesting that certain HMPV genotypes may have enhanced fitness [28]. Our results particularly complement Saikusa et al.’s [29] findings on HMPV genetic diversity, though we demonstrate a more pronounced shift towards A2b2 dominance than previously reported.

Geographic patterns, identified through rarefaction analysis, show significant regional structuring of viral diversity. Africa exhibits the highest rarefied diversity (3.00, 95% CI: 1.00–3.05) despite lower sampling intensity, while Asia shows distinct evolutionary patterns [30]. The recent detection of A2b2 in Europe (2023–2024) likely reflects both true viral circulation changes and shifts in surveillance strategies amid evolving post-pandemic respiratory virus monitoring [31]. These geographic patterns expand upon earlier work by Rodriguez et al. [32], who noted regional variation in HMPV evolution but lacked the quantitative framework to assess sampling bias. Our findings also challenge previous assumptions about HMPV’s global uniformity [33], suggesting instead that regional selective pressures may play a crucial role in viral evolution.

### Clinical–evolutionary correlations and disease severity

The directional evolution towards A2b2 dominance coincides with concerning clinical trends in HMPV-associated disease severity. Recent surveillance reports document substantial increases in HMPV-associated severe acute respiratory infections (SARI) in both paediatric and adult populations, particularly in China during 2024–2025, though direct correlation with A2b2 variants requires further investigation. Hospital-based surveillance studies suggest that HMPV infections may be associated with distinct clinical presentations, including prolonged viral shedding and increased rates of hospitalization among elderly patients. The temporal correlation between A2b2’s emergence in our genomic analysis and reports of increased HMPV clinical burden suggests potential epidemiological significance, though our genomic analysis cannot establish direct causation.

These clinical observations align with our genomic findings of A2b2’s selective advantage and suggest that evolutionary changes may correlate with altered pathogenic potential. This pattern aligns with observations in other respiratory viruses, where genetic evolution has been linked to altered clinical outcomes, particularly in vulnerable populations, including immunocompromised individuals and those with underlying respiratory conditions.

### G gene duplication mechanisms and immune evasion

The G gene duplications characteristic of A2b2 variants represent a critical evolutionary innovation with potential immunological implications. Similar to patterns observed in RSV evolution, where G protein modifications have been associated with increased viral fitness and antigenic drift [26], HMPV G gene duplications may facilitate immune evasion through altered antigenic surfaces. Experimental evidence from related pneumoviruses suggests that G gene duplications can alter neutralizing antibody recognition, potentially reducing the effectiveness of naturally acquired immunity.

The mechanistic basis for potential enhanced immune evasion may involve both steric hindrance of antibody binding sites and altered protein folding that could mask conserved epitopes. This evolutionary strategy represents a sophisticated adaptation that may balance immune evasion with viral fitness, potentially explaining A2b2’s successful global expansion. However, functional validation of these hypotheses requires targeted experimental studies beyond the scope of our genomic analysis.

### Public health implications

These findings have important public health implications that extend beyond traditional surveillance frameworks. The strong directional evolution of A2b2, particularly in relation to G gene duplications, mirrors patterns observed in RSV, where similar genetic modifications contributed to increased viral fitness and immune evasion [26, 27]. Real-time monitoring of such evolutionary changes is critical for predicting outbreak potential and assessing the effectiveness of vaccines and antiviral strategies.

Recent studies have shown that alterations in viral attachment proteins can significantly impact population susceptibility [34], suggesting that A2b2 variants with G gene duplications may require updates to current surveillance strategies. Additionally, the uneven geographic distribution of A2b2 – marked by its limited detection in Africa and North America despite its presence in other regions [11] – highlights the urgent need for enhanced global genomic surveillance networks [35].

The post-pandemic landscape has reinforced the value of early detection systems for respiratory viruses [36]. Our findings suggest that HMPV surveillance should be more comprehensively integrated into existing respiratory virus monitoring frameworks, particularly given reports of increasing clinical burden and the recent surge in severe cases globally. This is especially relevant in the context of co-circulating respiratory pathogens, where interactions between viruses can shape transmission dynamics and disease severity [37]. The quantitative framework we present offers a model for real-time assessment of viral evolution patterns, potentially enabling more rapid responses to emerging variants of concern.

The clinical implications of A2b2 evolution may extend to therapeutic and preventive strategies. Current antiviral approaches may need reassessment in light of A2b2’s altered molecular characteristics, and vaccine development efforts should consider the antigenic properties of dominant circulating variants. Furthermore, infection control measures in healthcare settings may require modification to account for the potentially enhanced transmission characteristics of A2b2 variants, though such modifications should be based on clinical evidence rather than genomic inference alone.

### Future directions

While our analytical approach accounts for key biases, surveillance limitations remain. The global sequencing database is still disproportionately weighted towards regions with stronger genomic infrastructure, potentially obscuring patterns in areas with limited surveillance [38]. Future studies should incorporate clinical and epidemiological data to assess whether A2b2’s genetic changes affect transmissibility, immune evasion, or disease severity.

Additionally, integrating functional studies will be critical to determining whether G gene duplications in A2b2 confer adaptive advantages. Strengthening genomic surveillance networks and expanding sequencing efforts in underrepresented regions will be crucial for tracking the evolution of HMPV and understanding its broader impact on respiratory infections.

Future research would benefit from the development of standardized analytical pipelines that can handle sampling bias in real time, potentially incorporating machine learning approaches to predict evolutionary trajectories [39]. International collaborative networks, like those established for influenza surveillance, could help address current geographic gaps and provide early warning of emerging variants [40]. Moreover, the integration of antigenic mapping techniques with genomic data could better characterize the immunological implications of A2b2 evolution, particularly important for vaccine development and therapeutic strategies.

Critical research priorities include longitudinal studies correlating A2b2 genetic markers with clinical outcomes, functional characterization of G protein variants in cell culture and animal models, and population-level serological surveys to assess the impact of antigenic evolution on herd immunity. Additionally, investigating the potential for A2b2 variants to evade existing diagnostic assays will be essential for maintaining effective surveillance systems.

### Limitations

Our study has several important limitations. First, sequence data from public repositories inherently reflect global surveillance inequities, with regions possessing robust genomic infrastructure contributing disproportionately to our dataset. While our statistical approaches account for sampling heterogeneity, evolutionary patterns in areas with limited sequencing capacity – particularly low- and middle-income countries – may be underrepresented.

Second, temporal heterogeneity in sampling intensity presents analytical challenges. The substantial increase in sequences during the Recent Phase likely reflects both enhanced surveillance efforts and true changes in viral prevalence. Although our entropy-based analysis and rarefaction approaches help disentangle these factors, they cannot completely resolve this temporal confounding.

Third, the lack of integrated clinical and epidemiological data constrains our ability to correlate genomic changes with phenotypic outcomes. The clinical correlations we discuss are based on concurrent literature and temporal associations rather than direct analysis of linked genomic–clinical datasets.

Fourth, our analysis lacks selection pressure assessment through dN/dS analysis and site-specific selection testing, which would provide deeper insights into the evolutionary forces driving A2b2 emergence and represents an important area for future investigation.

Finally, recent sequences (2023–2024) may reflect temporary evolutionary trajectories not yet subject to long-term selective pressures, particularly given the altered epidemiological landscape following COVID-19 pandemic interventions.

## Conclusion

Our analysis shows that the global rise of the HMPV A2b2 clade is driven by non-random evolutionary patterns rather than increased surveillance alone. Regional differences in viral diversity, particularly G gene duplications, suggest distinct evolutionary trajectories that warrant continued monitoring. These findings underscore the need for sustained genomic surveillance to track HMPV evolution and anticipate emerging variants. Future research integrating clinical and epidemiological data will be crucial for assessing the public health impact and guiding evidence-based surveillance strategies.

## Data Availability

The data used in this study were obtained from publicly available sources, including the Nextstrain database. All sequence data and associated meta-data are accessible through Nexstrain under standard terms of use.

## Figure 1. Long description

The x-axis is labeled Year, spanning from 1995 to 2025. The y-axis is labeled Value, ranging from 0 to 55. Series 1 is a blue solid line with circular markers, starting near zero in 1995, remaining low until 2000, then gradually rising with fluctuations and a sharp peak above 50 around 2017, followed by a steep drop and renewed variability through 2024. Series 2 is an orange dashed line, flat at value 18 from 1995 to 2007, then jumping to 52 in 2008, dropping to 18 in 2010, and oscillating between 40 and 52 from 2011 to 2024. The legend in the upper left identifies Series 1 and Series 2. Peaks in Series 2 do not always align with those in Series 1, indicating temporal heterogeneity between the two data sets.

Navigate back to Figure 1..

## Table 1. Long description

From top to bottom, the table lists Africa, Asia, Europe, North America, Oceania, and South America. Each region is divided into Early, Mid, and Recent phases. For Africa: Early phase has N 6, A2b2 percent 0, effect size not available; Mid phase N 46, 15.2 percent, effect size 0.67 (medium), rate of change 4.8 percent per year, phase p value 0.142, trend p value 0.006; Recent phase N 53, 47.8 percent, effect size 1.42 (large), phase p value 0.008. For Asia: Early phase N 12, 8.3 percent, effect size not available; Mid phase N 142, 52.1 percent, effect size 1.89 (large), rate of change 6.3 percent per year, phase p value 0.003, trend p value less than 0.001; Recent phase N 130, 71.5 percent, effect size 2.15 (large), phase p value less than 0.001, trend p value less than 0.001. For Europe: Early phase N 4, 0 percent, effect size not available; Mid phase N 68, 44.1 percent, effect size 1.54 (large), rate of change 5.7 percent per year, phase p value 0.024, trend p value less than 0.001; Recent phase N 82, 68.3 percent, effect size 1.78 (large), phase p value 0.002, trend p value 0.002. For North America: Early phase N 15, 6.7 percent, effect size not available; Mid phase N 126, 38.9 percent, effect size 1.12 (large), rate of change 4.2 percent per year, phase p value 0.089, trend p value 0.004; Recent phase N 115, 62.6 percent, effect size 1.45 (large), phase p value 0.012. For Oceania: Early phase N 3, 0 percent, effect size not available; Mid phase N 24, 33.3 percent, effect size 0.89 (large), rate of change 3.9 percent per year, phase p value 0.231, trend p value 0.018; Recent phase N 22, 59.1 percent, effect size 1.23 (large), phase p value 0.045, trend p value 0.045. For South America: Early phase N 4, 0 percent, effect size not available; Mid phase N 52, 42.3 percent, effect size 1.34 (large), rate of change 5.1 percent per year, phase p value 0.067, trend p value 0.003; Recent phase N 47, 66 percent, effect size 1.67 (large), phase p value 0.015. Statistically significant results are noted at alpha equals 0.05 after Benjamini–Hochberg correction. Time periods are defined as Early phase (2012 or earlier), Mid phase (2013 to 2018), and Recent phase (after 2018). Effect size is interpreted as small (0.2 to 0.5), medium (0.5 to 0.8), and large (greater than 0.8). Rate of change is the average annual increase in A2b2 prevalence.

Navigate back to Table 1..

## Figure 2. Long description

The x-axis is labeled Year, spanning from 2000 to 2020. The y-axis is labeled Proportion of Sequences, ranging from zero to three. Each vertical bar represents a year and is divided into colored segments by region: Africa (dark purple), Asia (blue), Europe (teal), North America (green), Oceania (light green), and South America (yellow), as indicated in the legend at the right. From 2000 to about 2010, only North America, Europe, and Oceania are represented, each with a single segment per year. After 2010, the bars become stacked with multiple regions per year, showing the addition of Africa, Asia, and South America. The height and composition of the bars vary, with some years dominated by a single region and others showing more equal contributions. The most recent years (2010 and 2020) display all six regions, with the segments for Africa, Asia, and South America appearing only in these later years. This pattern demonstrates that early sampling was limited to a few regions, while later years show broader geographic representation and increased sampling intensity, but with persistent regional disparities.

Navigate back to Figure 2..

## Figure 3. Long description

The x axis is labeled year, spanning from before 2000 through 2020. The y axis is labeled proportion, ranging from 0 to 3. Each vertical bar represents a time point and is divided into colored segments: A1 in purple, A2a in blue, A2b1 in green, and A2b2 in yellow. Early years show higher proportions of A1 and A2a, with A2b1 present throughout. From around 2010 onward, yellow segments representing A2b2 appear and increase in size, becoming dominant by 2020. Error bars extend vertically from each bar segment, indicating 95 percent confidence intervals. The legend at the right identifies clade colors. Sampling uncertainty, shown by the length of error bars, is greater in periods with fewer sequences, especially in early years.

Navigate back to Figure 3..
